# RIRS with Vacuum-Assisted Ureteral Access Sheath versus MPCNL for the Treatment of 2-4 cm Renal Stone

**DOI:** 10.1155/2020/8052013

**Published:** 2020-05-14

**Authors:** Dehui Lai, Yongzhong He, Xun Li, Meiling Chen, Xingrong Zeng

**Affiliations:** ^1^Urology, Fifth Affiliated Hospital, Guangzhou Medical University, Guangzhou, Guangdong, China; ^2^Minimally Invasive Technique and Product Translational Center, Guangzhou Medical University, Guangzhou, Guangdong, China; ^3^College of Materials Science and Engineering, Key Lab of Guangdong Province for High Property and Functional Polymer Materials, South China University of Technology, Guangzhou, China

## Abstract

**Objective:**

Comparison of outcomes between RIRS with vacuum-assisted ureteral access sheath (V-UAS) and MPCNL in the treatment of renal stone.

**Materials and Methods:**

28 patients with 2-4 cm renal stone were treated using RIRS with 14/16 F V-UAS. The outcomes were compared to those who underwent MPCNL with 16 F Amplatz sheath using a matched-pair analysis in a 1 : 2 scenario. Matching criteria included stone size, location and laterality, gender, age, BMI, and degree of hydronephrosis. Patients' demographics, perioperative and postoperative characteristics, complications, stone-free rate (SFR), and auxiliary procedures were compared.

**Results:**

Mean operative times for the RIRS and MPCNL groups were 72.4 ± 21.3 minutes and 67.4 ± 25 minutes (*P* = 0.042). Postoperative pain was significantly less in the RIRS group. The initial SFR was 50% for the RIRS group and 73.2% for the MPCNL group (*P* = 0.035). The final SFR at postoperative three months improved to 89.3% for the RIRS group and 92.9% for the MPCNL group (*P* = 0.681). The auxiliary procedure rates were higher in the RIRS group (42.9% vs. 25%, *P* = 0.095). The overall complication rate in the RIRS group was lower, but the significant difference was not found.

**Conclusion:**

In the treatment of 2-4 cm renal stone, using V-UAS in RIRS can improve surgical efficiency with lower postoperative early pain scores. Comparing with MPCNL, its initial SFR was more depressed, and there is still a trend towards requiring more auxiliary procedures to achieve comparable final SFR.

## 1. Introduction

Minimally invasive percutaneous nephrolithotomy (MPCNL) is a reasonable treatment for 2-4 cm renal stones with a high success rate and low morbidity [1, 2]. It can achieve a stone-free rate (SFR) of 78%-95% [2, 3]. However, MPCNL is still associated with significant potentially serious complications, such as hemorrhage, urosepsis, hydrothorax, urinary leakage, or even death.

In the recent decade, retrograde intrarenal surgery (RIRS) has been used in some centers for the more complex stones. RIRS has reported comparable SFR with low complications. Takazawa et al. reported 100% stone clearance using RIRS for 2-4 cm renal stones [4]. Riley et al. showed a 90.9% success rate for stones averaging three centimeters in size [5]. The overall complication rates of RIRS for 2-4 cm renal stones vary from 11.7 to 37.2% [4–9]; most of the complications are minor.

The shortages of RIRS in treating large stone are time-consuming and potential pyelovenous backflow due to promoted intrarenal pressure results from outflow obstructed by tiny fragments. Recently, a novel vacuum-assisted ureteral access sheath (V-UAS, ClearPetra, Well Lead Medical, China) was introduced into the urological department.

**Figure 1:**
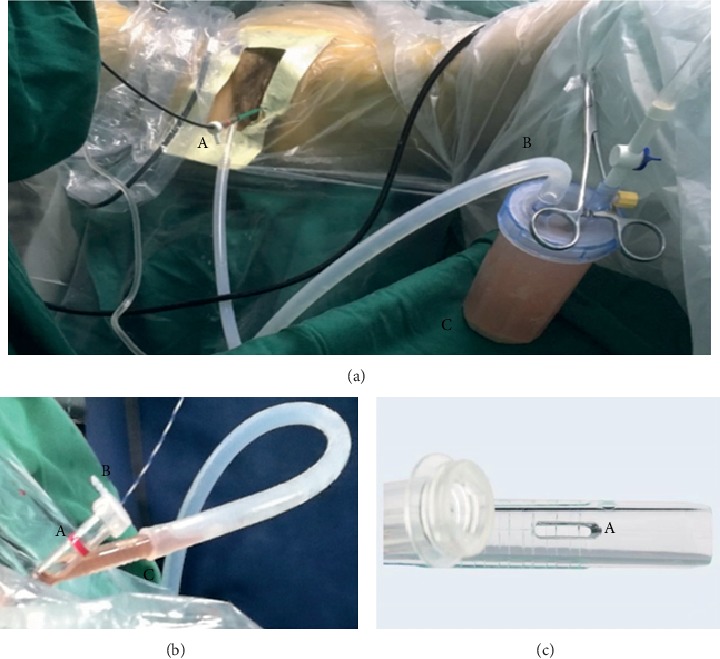
(a) Sucking out the tiny fragments and dust during lithotripsy by V-UAS: (A) inserting a flexible endoscope into the sheath through the aperture of rubber cap to gain a closed system, (B) the oblique drainage tube was connected to a negative pressure aspirator, and (C) a stone collection bottle is connecting the UAS and the negative pressure suction. (b) Structure of V-UAS: (A) straight introduced tube with a red marker, (B) rubber cap, and (C) oblique drainage tube. (c) Structure of oblique drainage tube in V-UAS: (A) a longitudinal slit on this oblique drainage tube that is a pressure-regulating vent.

This novel UAS differs from the conventional one by having an oblique drainage tube that is constructed as a handle (Figure 1). The oblique drainage tube was connected to a negative pressure aspirator during the surgery. There is a longitudinal slit on this oblique drainage tube that is a pressure-regulating vent. A stone collection bottle is connecting the UAS and the negative pressure suction. A rubber cap with a central aperture is used to cover the straight end of the handle to gain a closed system, even inserting a flexible endoscope into the sheath through the aperture. Irrigation fluid and tiny fragments are aspirated in the gap between the scope and the UAS and then go through the oblique drainage tube. When plenty of stone fragments are sucked into the UAS that partially obstructed the outflow or operative vision was cloudy by lots of dust, withdrawing the scope till the red line of the straight tube will open up an unimpeded passage to aspirate the stone. Pressing the slit can increase the suction power. This V-UAS has the potential to suck out the tiny fragments and dust during lithotripsy due to its simultaneous suction property with continuous irrigation and guarantee clear vision, as well as unobstructed outflow passage. The use of this UAS may be considered as a new way to improve the efficacy of RIRS.

In this study, we compare the outcomes between RIRS with V-UAS and MPCNL in the treatment of 2-4 cm renal stone using a matched-paired analysis. To our knowledge, this is the first paper to report the clinical data of this device.

## 2. Material and Methods

The digital files of 153 patients who were treated for 2-4 cm renal stone between July 2017 and July 2018 were reviewed, and a database was constructed. Patients with a congenital renal anomaly, ureteropelvic junction obstruction, ureteral stricture, previous surgery, refractory infection, and pyonephrosis were excluded. It resulted in 28 patients who underwent RIRS which were assigned to group A. A matched group of 56 patients who underwent MPCNL in the same period was identified and assigned as group B. Matching criteria included stone size, location and laterality of the stones, gender, age, body mass index, and degree of hydronephrosis. The 2 : 1 ratio was chosen due to the larger number of patients who had undergone MPCNL.

Stone size and location were assessed preoperatively by noncontrast CT scan. Stone size was measured in its largest diameter. The stone burden was defined by its surface area and was calculated following the European Association of Urology guidelines [10]. Stone clearance was defined as the absence of any fragments by low-dose noncontrast CT. Preoperative laboratory tests included routine CBC, urine analysis, urine culture, serum creatinine estimation, and coagulation studies.

All procedures were performed under continuous epidural anesthesia. Parenteral prophylactic antibiotics were administrated to all the patients with a negative preoperative urine culture. Patients with positive urine cultures were treated with appropriate antibiotics until the infection was under control. Patients scheduled to undergo RIRS had double J stents (D-J) placed in outpatient surgery 7-10 days before the RIRS surgery.

### 2.1. MPCNL Technique

Under adequate anesthesia, a 5 Fr. ureteral catheter was first inserted into the affected ureter in the lithotomy position. The patient was then turned into a prone position with a pillow under the upper abdomen. Renal puncture of the targeted calyx was performed using fluoroscopic guidance with an 18-gauge needle. Access is generally gained through a posterior calyx using the “bull's-eye” technique. Once the needle was properly placed, a 0.035-inch guidewire was inserted through the needle shaft and advanced into the collecting system. Serial tract dilatation was accomplished using Amplatz dilators starting at 8 Fr. and extending up to 16 Fr. Next, a matched size access sheath was advanced into the collecting system. The stones were fragmented using either a holmium laser or a pneumatic lithotripter through an 8.5/12 Fr. rigid mini-nephroscope (Richard Wolf, Germany). The larger fragments were removed with forceps, and the smaller pieces were flushed out using a pulsed perfusion pump. A 6 Fr. D-J was inserted in antegrade fashion over a guidewire, and a balloon nephrostomy tube was inserted through the nephrostomy sheath at the end of the procedure. Low-dose renal CT was routinely performed on postoperative day one to assess the residual stone. Patients with significant remaining fragments underwent auxiliary procedures on the fifth to seventh postoperative days. These included second-look MPCNL, RIRS, or both. The nephrostomy tube was removed when the drainage was grossly clear, and the patient was discharged the next day.

### 2.2. RIRS Techniques

RIRS was performed in the dorsal lithotomy position. After D-J stent retrieval, a retrograde pyelography was performed. Next, a 0.035-inch guidewire was introduced into the upper tract. A 14/16 Fr. V-UAS was inserted over the guidewire. A 9.9 F digital flexible ureteroscope (URF-V, Olympus) was advanced over the guidewire and into the renal pelvis. A complete inspection of the entire collecting system was performed. Large stones were fragmented with Flexiva 200 *μ*m holmium laser fibers (Boston Scientific). An energy setting of 1-1.5 Joule and a rate of 15-20 Hertz were generally used. Larger fragments were removed using the stone basket. Smaller fragments were sucked out as far as possible. The rest was left in situ for spontaneous passage. At the end of the procedure, the collecting system was reinspected both visually and fluoroscopically for any large stone fragments. UAS was removed along with the ureteroscope. The ureteral injury was visually assessed and documented at this time. A 6 F D-J was placed in all patients at the end of the procedure. Patients were discharged the next day.

Patients were assessed by low-dose CT on postoperative day 1. In patients with significant residual stones, a second-stage RIRS was performed. D-J was removed 2-4 weeks postoperatively.

Final SFR was assessed with low-dose noncontrast CT in all the patients 3 months after the procedure. A visual analogue pain scale (VAS) was used to quantify the degree of pain. Patients' demographics, perioperative and postoperative characteristics, complications, hemoglobin drop, patients' VAS, length of hospitalization, SFR, and auxiliary and total number of procedures were compared between the RIRS and MPCNL groups.

Statistical analysis was performed using the SPSS 22.0® software. Continuous variables were compared using the Student *t* and Wilcoxon test. Univariable analysis was conducted using the Pearson *χ*^2^ statistic or Fisher's exact test for categorical data. *P* values < 0.05 were considered statistically significant.

## 3. Results

Demographics and preoperative data are shown in Table 1. They were comparable in these two groups. Positive urine cultures were found in eight patients in the RIRS group and 21 in the MPCNL group. All of the infections were successfully treated using appropriate antibiotics.

Perioperative and postoperative data are displayed in Table 2. Mean operative times for the RIRS and MPCNL groups were 72.4 ± 21.3 minutes (range 42-106) and 67.4 ± 25 minutes (range 44-114), respectively, *P* = 0.042. Mean fluoroscopy time was significantly shorter for the RIRS group (1.6 ± 0.5 vs. 4.4 ± 2.1 minutes, *P* < 0.001). Mean drop in the postoperative hemoglobin level was 0.5 ± 0.21 (range 0.1-0.7) g/dL in the RIRS group, which was found to be statistically less (*P* < 0.001) than the corresponding decrease of 1.9 ± 1.3 g/dL (range 0.5-4) in the MPCNL group. Moreover, postoperative pain was significantly less in the RIRS group.

The overall complication rate in the RIRS group was lower; however, the difference was not statistically significant. Five patients in the MPCNL group experienced postoperative fever that required antipyretics, whereas two patients in the RIRS group had similar complications (Clavien grade I). One patient in the RIRS group and three patients in the MPCNL group had emesis (Clavien grade I). They were successfully treated with an antiemetic. Urosepsis was encountered in one patient who underwent RIRS and in two of the MPCNL patients. They were all successfully treated with appropriate intravenous antibiotics and resuscitation (Clavien II). Blood transfusion was required for two patients in the MPCNL group (Clavien II) but none in the RIRS group. Ureteral perforation (Clavien IIIa) occurred in one patient in the RIRS group and one in the MPCNL group. They were successfully treated with indwelling D-J for eight weeks without sequelae. In addition, one patient in the RIRS group developed steinstrasse and required rigid ureteroscopic intervention (Clavien IIIb).

The initial SFR was 50% for the RIRS group and 73.2% for the MPCNL group (*P* = 0.035). 12 of the 14 post-RIRS patients required a second-stage RIRS. Second-stage RIRS was not attempted in two patients due to the inaccessible lower calyx containing the stones noted during the first RIRS. One patient in MPCNL did not agree to do a second operation. Second-look MPCNL combined with RIRS was required for 14 post-MPCNL patients. The final SFR improved to 89.3% for the RIRS group and 92.9% for the MPCNL group (*P* = 0.681). The auxiliary procedure rates were higher in the RIRS group (42.9% vs. 25%), but significant difference was not found. The stone analysis revealed that calcium stones accounted for 60.7% of the stones in the RIRS group and 69.6% in the MPCNL group. Struvite stones were the next most commonly found stones. There were no statistically significant differences noted in the stone compositions between the two groups.

## 4. Discussion

The EUA guidelines of 2016 established PCNL as the primary treatment for calculi greater than 2 cm [3]. MPCNL is effective for managing these stones with comparable SFR and operative time to conventional PCNL with the merit of higher safety due to a lower rate of bleeding [11, 12]. Even though the efficacy of MPCNL is well-recognized, it is still associated with some serious complications.

On the other hand, treating larger renal calculi with RIRS is tedious and time-consuming. It may increase the risk of sepsis [13]. It can promote intrarenal pressure results from outflow obstructed by tiny fragments. Although with the improvement in flexible endoscopes, the accessories, and the techniques, RIRS has been reported as a feasible alternative for larger renal stone with fewer complications [14]. There was inadequate data to validate the decision process. As V-UAS has the potential to suck out the tiny fragments and dust during lithotripsy due to its simultaneous suction property with continuous irrigation and guarantee clear vision, as well as unobstructed outflow passage, RIRS has been performed sporadically at our institution for large stone burden. To our knowledge, this is the first paper to compare the clinical outcome between RIRS with V-UAS and MPCNL.

It has been shown that the overall SFR for RIRS ranged from 77% to 93% after auxiliary procedures for renal stone > 2 cm [5, 7–9, 13–16] were performed. Riley et al. showed an overall SFR of 90.9% when performing staged RIRS on 22 patients with renal stones > 3 cm, except those with complete staghorn calculi [5]. Similarly, Cohen et al. [17] performed staged RIRS on 36 patients with staghorn calculi with an average size of 3.7 cm. They achieved an initial SFR of 81%. As far as we know, only four previous studies [9, 18–20] compared the SFR of PNCL and RIRS with conventional UAS in the management of the larger renal stone burden. Among them, initial SFR of the PCNL group after a single procedure was significantly higher. Final SFR was shown in 3 studies which were comparable in both groups. Undoubtedly, PCNL is direct and energetic method in the first stage. Although using V-UAS in our research, the initial SFR of the RIRS group was still significantly lower than that of the MPCNL group—50% vs. 73.2%. Interestingly, the final SFR after auxiliary procedures was similar in both groups. There was no difference in auxiliary procedure rate between the two groups. However, the RIRS group had 42.9% versus only 25% in the MPCNL group. Moreover, caution must be taken in that each case in the RIRS group was prestented. Auxiliary procedures here are defined as the additional procedures which are needed to deal with the rest stone and complications. We did not calculate prior D-J stenting as an auxiliary procedure. However, there is still a trend towards requiring more auxiliary procedures in the RIRS group to achieve comparable final SFR with the MPCNL group.

Besides the size, the location of the stone is another crucial factor affecting the SFR of RIRS. Cohen et al. [17] compared the SFR of the different positions of renal stones in patients who underwent RIRS. They found that the lowest SFR was observed in the lower pole stone. Resorlu et al. [21] demonstrated that the presence of a lower pole infundibulopelvic angle (IPA) > 45° is associated with higher RIRS success rate. RIRS could not be performed for the lower pole stones in two of our patients due to inaccessibility of the lower pole calyx. The IPA for these two calyces was 38° and 40°. Therefore, IPA should be measured before performing RIRS in patients with lower pole staghorn stones. In patients with IPA < 45°, MPCNL may be a more appropriate choice. Moreover, the initial and final SFR in the RIRS group with V-UAS are comparable with the previous study of using conventional UAS during RIRS. So far, no evidence is showed that using V-UAS in RIRS will increase the SFR.

Due to the less-invasive nature of RIRS and the suction technique of using V-UAS, as well as the renal parenchyma injury while establishing percutaneous renal access of MPCNL, the complication rate of RIRS is expected to be lower than that of MPCNL. However, the difference in severity was not found to be significant per the modified Clavien grade. The use of a small access tract in MPCNL in this study might decrease the risk of bleeding and reduce the transfusion rate. Only two patients (3.6%) required a blood transfusion in the MPCNL group. There is no significant difference in both groups.

The infectious complication rate of RIRS varied from 1.7% to 18.8% [22, 23]. The incidence of fever of MPCNL ranged between 0% and 32.1% [24]. Prolonged operative time, high intrarenal pressure, and preoperative urine infection are also known risk factors for postoperative fever and urosepsis, especially in treating staghorn calculi [25]. Due to the larger burden, long operative times were recorded for both groups. Mean operation time for the RIRS and MPCNL groups was 72.4 ± 21.3 and 67.4 ± 25 minutes, respectively (*P* = 0.042). However, dramatically, the operating times for RIRS with V-UAS were found to be shorter than those reported by El-Anany et al. [15], who performed RIRS with conventional UAS. Using V-UAS has the potential to suck out the tiny fragments and dust during lithotripsy with continuous irrigation and guarantee clear vision. It will significantly decrease the operative time. On the other hand, the operation time of MPCNL was comparable with the previous study [18–20].

Akman et al. [26] demonstrated that excessive intrarenal pressure in RIRS could lead to intrarenal reflux. Schwalb et al. [27] found that high-pressure irrigation in RIRS leads to renal extravasation. In this study, preoperative positive urine culture was shown in 28.6% of patients in the RIRS group and in 37.5% in the MPCNL group. We just had two fever and one urosepsis in the RIRS group and five fever and two urosepsis in the MPCNL group. They were not statistically significant. However, the infectious complication rate of RIRS with V-UAS in this cohort was comparable with that in the study without or with less infection case [4–9, 18–20]. The use of V-UAS, which has the suction function to balance the irrigation and outflow, may contribute to the low rate of high-intrarenal pressure-associated complication.

UAS is used to allow repeat passage of ureteroscope and passive egress of irrigation fluid and stone fragments. However, several studies have shown that the overdistention UAS may result in ureteral ischemia and wall injuries, which may progress to ureteral perforation and stricture [28]. In this study, all RIRS patients were prestented for 7-10 days to allow passive ureteral dilation and to minimize the risk of possible ureteral injury. Also, due to the large stone burden in our patients, RIRS with V-UAS procedures tended to be quite long with a mean time of over 70 minutes. Only one ureteral perforation was observed and successfully treated with D-J for eight weeks with no sequelae. It is comparable with other studies which were undergoing RIRS with conventional UAS [15–20, 29].

Steinstrasse is another reported problem in patients with large stone burden who underwent RIRS. Mariani [14] reported that 18.7% of patients with renal stone > 4 cm after RIRS developed steinstrasse. Traditional options for reducing the fragment burden in RIRS include active extraction with a basket through a ureteral access sheath or use of a “dusting” technique in which the patients pass the tiny fragments spontaneously over time. Using V-UAS can suck out the tiny fragments and dust with continuous irrigation besides these two methods. Also, it can reduce the numbers of fragment residuals. Only one case steinstrasse (3.5%) was found in this study and successfully treated with the auxiliary ureteroscopic procedure.

Our RIRS patients had shorter fluoroscopic time than the MPCNL patients. It is consistent with other reports; more fluoroscopic time is generally required for establishing the nephrostomy tract. Another advantage of RIRS was the shorter hospital stay. RIRS patients generally experience less pain, have less blood loss, and tend to recover faster. However, due to this nation's health insurance reimbursement policy, RIRS must be performed as inpatient surgery; thus, the hospital stays in RIRS patients in this study tend to be longer than in most other reports.

Due to the large residual stone sizes and high incidence of positive urine cultures and/or infected stones, SWL was not utilized as an auxiliary procedure.

The main limitation of this study is its retrospective study with a relatively small number of patients and nonrandomized design. A multicenter randomized study with a larger sample size and a longer follow-up time would be ideal. Another limitation was that we did not measure the intrarenal pelvic pressure during the procedure to get more information.

## 5. Conclusion

In the treatment of 2-4 cm renal stone, using V-UAS in RIRS can improve surgical efficiency with lower postoperative early pain scores. Comparing with MPCNL, its initial SFR was more depressed, and there is still a trend towards requiring more auxiliary procedures to achieve comparable final SFR.

## Figures and Tables

**Table 1:** Demographic data of patients.

Variable	Group A (RIRS)	Group B (MPCNL)	*P* value
	28 patients	56 patients	
Age (year), mean (SD), range	45.2 (10.4), 21-65	49.6 (12.2), 23-72	0.296
Gender (males/females)	16/12	26/30	0.355
BMI (kg/m^2^), mean (SD), range	24.98 (3.51), 19-32	25.32 (4.12), 20-33	0.104
Stone-affected side (left/right)	13/15	30/26	0.537
Grade of hydronephrosis (no.)			0.061
None	16	20	
Mild	12	36	
Charlson comorbidity index			0.864
0 (%)	13	24	
1 (%)	10	18	
2 (%)	3	10	
3 (%)	2	4	
Renal stone location, no. (%)			0.562
Renal pelvic+upper pole	7	17	
Renal pelvic+middle pole	4	12	
Renal pelvic+lower pole	6	11	
Renal pelvic+middle pole+lower pole	5	11	
Renal pelvic+upper pole+middle pole	6	5	
Largest stone size (mm), mean (SD), range	35.3 (6.3), 25-39	38.2 (5.4), 28-40	0.074
Renal stone burden (mm^2^), mean (SD), range	676.1 (42.2), 391.2-803.4	729 (83.7), 412.3-843.2	0.089
Stone density (HU), mean (SD), range	894.3 (232.3), 650-1103	845.2 (240.2), 600-1206	0.43
Positive urine culture, no. (%)	8 (28.6%)	21 (37.5%)	0.417

**Table 2:** Perioperative and postoperative data of patients.

Variable	Group A (RIRS)	Group B (MPCNL)	*P* value
	28 patients	56 patients	
Fluoroscopy time (min), mean (SD), range	1.6 (0.5), 0.8-3.5	4.4 (2.1), 2-10	<0.001
Operative time (min), mean (SD), range	72.4 (21.3), 42-106	67.4 (25), 44-114	0.042
Hospitalization stay (days), mean (SD), range	4.3 (2.9), 2-10	6.1 (3.2), 2-20	<0.001
Hemoglobin drop (g/dL), mean (SD)	0.5 (0.21), 0.1-0.7	1.9 (1.3), 0.5-4	<0.001
Complication (modified Clavien classification), no. (%)	6 (21.4%)	13 (23.2%)	0.854
Grade I	3	8	
Fever	2	5	
Emesis	1	3	
Grade II	1	4	
Infection	1	2	
Blood transfusion	0	2	
Grade IIIa	1	1	
Perforation	1	1	
Grade IIIb	1	0	
Steinstrasse	1	0	
Pain visual analogue score (1–10), mean (SD), range			
At 6 h	3.3 (1.3), 2-5	5.4 (1.1), 4-8	<0.001
At 24 h	2.0 (0.9), 2-5	4.2 (1.2), 3-8	<0.001
At 48 h	1.1 (0.3), 1-3	2.8 (1.4), 1-5	<0.001
Postoperative analgesics (diclofenac sodium), no. (%)	4 (14.3%)	23 (41.1%)	0.013
Initial stone-free rate, no. (%)	14 (50%)	41 (73.2%)	0.035
Final stone-free rate, no. (%)	25 (89.3%)	52 (92.9%)	0.681
Residual size (mm), mean (SD), range	21.1 (1.3), 15-22	25.1 (2.4), 13-23	0.06
Auxiliary procedures, no. (%)	12 (42.9%)	14 (25%)	0.095
Second-stage RIRS	12		
Second-look PCNL+RIRS		14	
Procedure per patient, mean (SD), range	1.43 (0.48), 1-2	1.25 (0.44), 1-2	0.255
Stone composition, no. (%)			0.8
Calcium oxalate	11 (39.3%)	28 (50%)	
Calcium oxalate and phosphate	6 (21.4%)	11 (19.6%)	
Uric acid	4 (14.3%)	7 (12.5%)	
Struvite	7 (25%)	10 (17.9%)	

## Data Availability

The data used to support the findings of this study are available from the corresponding author upon request.

## Abbreviations

RIRS: Retrograde intrarenal surgery
MPCNL: Minimally invasive percutaneous nephrolithotomy
V-UAS: Vacuum-assisted ureteral access
SFR: Stone-free rate
CIRF: Clinically insignificant residual fragments
VAS: Visual analogue pain scale
UAS: Ureteral access sheath
KUB: Kidney-ureter-bladder.
